# The Mitochondrial Blueprint: Unlocking Secondary Metabolite Production

**DOI:** 10.3390/metabo14120711

**Published:** 2024-12-18

**Authors:** Yang Li, Yujia Zhang, Xinyu He, Ziyi Guo, Ning Yang, Guohui Bai, Juanjuan Zhao, Delin Xu

**Affiliations:** 1 Department of Medical Instrumental Analysis, Zunyi Medical University, Zunyi 563099, China; liyang20040226@163.com (Y.L.); zhangyujia_zmu@163.com (Y.Z.); 19815319788@163.com (X.H.); guoziyi0521@163.com (Z.G.); 15077823203@163.com (N.Y.); 2 Department of Cell Biology, Zunyi Medical University, Zunyi 563099, China; 3 Department of Immunology, Zunyi Medical University, Zunyi 563099, China

**Keywords:** secondary metabolites, biosynthesis, mitochondria, regulation mechanism, signaling pathway

## Abstract

Mitochondrial metabolism plays a pivotal role in regulating the synthesis of secondary metabolites, which are crucial for the survival and adaptation of organisms. These metabolites are synthesized during specific growth stages or in response to environmental stress, reflecting the organism’s ability to adapt to changing conditions. Mitochondria, while primarily known for their role in energy production, directly regulate secondary metabolite biosynthesis by providing essential precursor molecules, energy, and reducing equivalents necessary for metabolic reactions. Furthermore, they indirectly influence secondary metabolism through intricate signaling pathways, including reactive oxygen species (ROS), metabolites, and redox signaling, which modulate various metabolic processes. This review explores recent advances in understanding the molecular mechanisms governing mitochondrial metabolism and their regulatory roles in secondary metabolite biosynthesis, which highlights the involvement of transcription factors, small RNAs, and post-translational mitochondrial modifications in shaping these processes. By integrating current insights, it aims to inspire future research into mitochondrial regulatory mechanisms in *Arabidopsis thaliana*, *Solanum tuberosum*, *Nicotiana tabacum*, and others that may enhance their secondary metabolite production. A deeper understanding of the roles of mitochondria in secondary metabolism could contribute to the development of new approaches in biotechnology applications.

## 1. Introduction

Secondary metabolites are a class of non-essential compounds produced by plants, such as *Arabidopsis thaliana*, *Nicotiana tabacum*, and *Solanum tuberosum*. These metabolites primarily encompass terpenes, phenols, alkaloids, and glycosides. Unlike primary metabolites, secondary metabolites are typically biosynthesized under adverse conditions, such as environmental stress [1]. This unique characteristic endows secondary metabolites with significant physiological and ecological functions, including participating in defense reactions, promoting growth, and acting as signaling molecules [2]. These same properties that make secondary metabolites beneficial to plants also make them potentially beneficial to humans. Mounts of secondary metabolites have also been found to possess great pharmacological and therapeutic potential for humans, with many exhibiting potent antioxidant, antimicrobial, and anticancer properties [3,4]. Therefore, secondary metabolites play a crucial role in the natural world and human health. However, due to their complex chemical structure, secondary metabolites’ biosynthesis is often controlled by a complex enzyme catalytic and regulatory system. Investigation of synthetic and regulatory mechanisms has been a popular research topic for a long time.

Mitochondria are ubiquitous organelles in plant cells and serve as central hubs for both degradative and biosynthetic processes. They not only facilitate various metabolic reactions, including fatty acid synthesis, the tricarboxylic acid cycle (TCA cycle), and acetate oxidation, but also produce a variety of signaling molecules to regulate cellular metabolism. Recent research has demonstrated that mitochondria are highly flexible and adaptable in metabolic regulation. For example, when potato tubers are exposed to pathogen-induced stress, mitochondria regulate the synthesis of solanine by altering their metabolic substrates [5]. Past research has mainly focused on the role of mitochondria in primary metabolism. However, recent studies have revealed that mitochondria also participate in the biosynthesis of secondary metabolites, which is a key response of organisms to environmental challenges. One study found that increased mitochondrial activity correlates with elevated secondary metabolite concentrations in *Arabidopsis thaliana* [6]. As metabolic hubs, the importance of mitochondria to secondary metabolism is evident. However, no comprehensive report on this topic is currently available; therefore, this paper reviews the recent studies on the regulation of secondary metabolite synthesis by mitochondria.

Past studies have focused on the relationship between mitochondria and primary metabolism. Extensive research has often focused on the relationship between the mitochondrial TCA cycle and plant photosynthesis and photorespiration [7], glycolysis [8], and amino acid metabolism [9] or how other mitochondrial metabolic pathways participate in plant primary metabolism. However, the role of mitochondria in secondary metabolite biosynthesis remains poorly understood. As the center of metabolism, mitochondria are closely related to secondary metabolite synthesis, particularly in plants [10]. Therefore, this paper aims to elucidate how mitochondria link primary and secondary metabolism, thus deepening our understanding of the role of mitochondria in metabolism. By revealing the mechanisms through which mitochondria affect the biosynthesis of secondary metabolites, this study provides novel insights and strategies for optimizing the production of secondary metabolites using mitochondrial regulation techniques.

## 2. Survey Methodology

### 2.1. Information Search Methodology

Firstly, the databases searched in this study include PubMed, Google Scholar, and Web of Science. The search terms used were “mitochondria”; “secondary metabolites”; “metabolic pathways”; “plant physiology”; “stress response”; “secondary metabolism”; and “ROS”. Secondly, the retrieval process was not limited by date or language, with the retrieval date set as October 2024. Articles were collected, primarily consisting of original research articles and reviews. To ensure the reliability of the data collection process, two independent reviewers were assigned to analyze the abstracts and main contents of these articles. They worked independently, and any discrepancies in their selections were resolved through discussion. Additionally, references from relevant major articles were reviewed to identify further pertinent publications. No automation tools were used in the data collection process, as the selection and analysis were conducted manually by the reviewers.

### 2.2. Inclusion and Exclusion Criteria for Information

To ensure the relevance and quality of the included studies, the following inclusion criteria were established: (1) studies conducted within a specified timeframe to ensure the timeliness and applicability of the results; (2) a clear definition of sample size to ensure the generalizability and validity of the findings; (3) explicit criteria for sample selection and processing, along with intervention and control measures, to assess the specific role of mitochondria in secondary metabolite synthesis. The exclusion criteria included the following: (1) duplicate studies, which refer to the same research appearing in multiple publications; (2) unreliable or insufficient data that may hinder the understanding of the relationship between mitochondrial function and secondary metabolite synthesis.

## 3. Mitochondrial Direct Regulation of Secondary Metabolism

**Figure 1:**
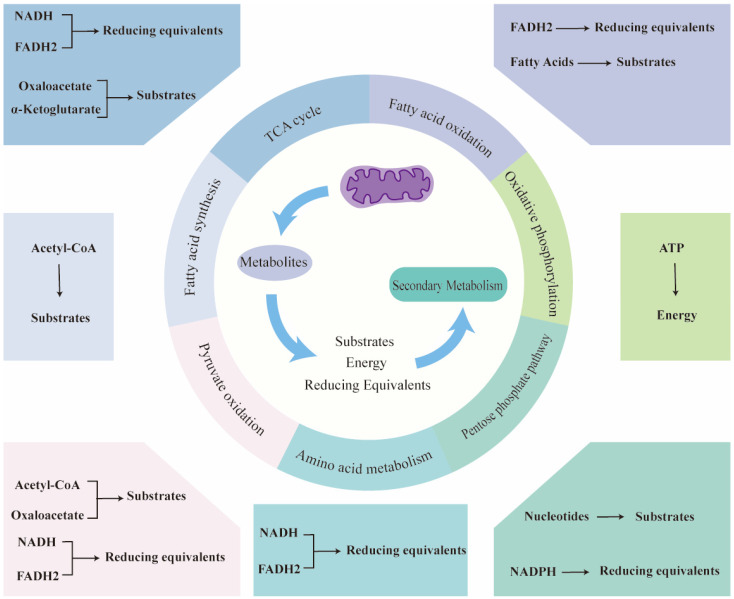
Mitochondrial direct regulation of secondary metabolism in plants. TCA = tricarboxylic acid cycle; NADH = nicotinamide adenine dinucleotide; FADH2 = flavin adenine dinucleotide; ATP = adenosine triphosphate; NADPH = nicotinamide adenine dinucleotide phosphate.

Mitochondria serve not only as the central hub for cellular energy metabolism but also play a crucial role in the biosynthesis of secondary metabolites in plants. Mitochondria directly influence the synthesis of these metabolites by providing essential precursor molecules, generating energy, and reducing equivalents (Figure 1). Figure 1 illustrates the mitochondrial key pathways that link primary and secondary metabolism in plants. This regulatory mechanism is of significant importance for plants, enabling them to respond effectively to environmental stressors and to modulate their physiological states.

### 3.1. Provision of Precursor Molecules

Mitochondria are pivotal nodes in various metabolic pathways, possessing the capacity to synthesize a range of essential precursor molecules that could serve as the foundation for the biosynthesis of secondary metabolites. Table 1 summarizes the primary metabolic pathways occurring in mitochondria, including the TCA cycle, pyruvate oxidation, the electron transport chain (ETC), oxidative phosphorylation, and fatty acid oxidation. Through these metabolic pathways, mitochondria not only supply energy to the cell but also have the potential to provide essential substrates for the production of secondary metabolites. As shown in Table 1, these potential substrates include key intermediates from these metabolic pathways, such as acetyl-CoA, oxaloacetate, and α-ketoglutarate, which are crucial for various secondary metabolite synthesis. Specifically, the acetyl-CoA produced from fatty acid oxidation and pyruvate oxidation within mitochondria can serve as a key substrate for the synthesis of carotenoids [11], sterols [12], and flavonoids [13]. The fatty acid oxidation process occurring within mitochondria produces fatty acids, which are critical components of membrane structures and can also be converted into carotenoids [14]. Mitochondria could also contribute to the synthesis of secondary metabolites by supplying necessary substrates through pathways such as the ETC and the pentose phosphate pathway.

### 3.2. Generation of Energy and Reducing Equivalents

Secondary metabolism often requires substantial amounts of energy and reducing equivalents, with mitochondria serving as the primary source of these resources. Mitochondria efficiently synthesize adenosine triphosphate (ATP) via oxidative phosphorylation, providing the necessary energy for various intracellular biosynthetic reactions, including those for secondary metabolite biosynthesis. For instance, the biosynthesis of flavonoids involves the metabolism of phenylalanine. It proceeds through enzymatic reactions, including phenylalanine hydroxylation, cinnamic acid transformations, and reduction reactions, all of which require energy input [24]. The synthesis processes of secondary metabolites such as triterpenoids, carotene, and anthocyanins are closely related to the energy state in the cell.

In addition to ATP, mitochondria generate NADH and FADH2 through the TCA cycle, amino acid metabolism, pyruvate oxidation within their matrix, and fatty acid oxidation. These reducing equivalents participate in a wide range of biosynthetic reactions, including hydroxylation, reduction, and decarboxylation, particularly in steps where reducing equivalents are needed to convert an intermediate. For example, the NADPH produced by the mitochondria through the pentose phosphate pathway can provide reducing equivalents for the synthesis of isopentenyl pyrophosphate and dimethylallyl pyrophosphate, which are subsequently used for isoprenoid biosynthesis [25]. In the flavonoid biosynthetic pathway, NADPH acts as an electron donor and participates in the chalcone formation reaction catalyzed by chalcone synthetase [24]. NADH is involved in reductive reactions during the biosynthesis of carotenoids, anthocyanins, and terpenes, among other secondary metabolites.

## 4. Mitochondrial Signaling Pathways for Regulating Secondary Metabolism

**Figure 2:**
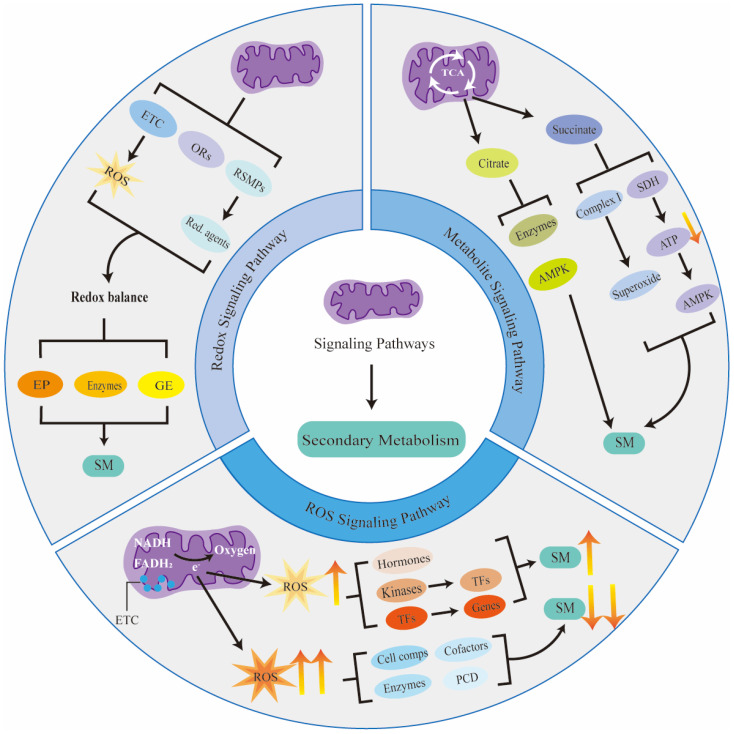
Mitochondrial signaling pathways regulating secondary metabolism in plants. SM = secondary metabolism; ETC = electron transport chain; ROS = reactive oxygen species; ORs = oxidoreductases; RSMPs = redox-sensitive metabolic pathways; Red. agents = reducing agents; EP = energy production; GE = gene expression; TCA = tricarboxylic acid cycle; AMPK = AMP-activated protein kinase; SDH = succinate dehydrogenase complex; ATP = adenosine triphosphate; TFs = transcription factors; Cell comps = cellular components; RCD = programmed cell death.

Mitochondria can sense changes in the intracellular and extracellular environment. To be more specific, mitochondrial activity can be influenced by the plant’s response to environmental changes, which often leads to an increase in mitochondrial metabolites, such as ROS. For instance, as the temperature rises within a certain range, mitochondrial ROS production will be increased in plants’ seeds [26]. These metabolites can then act as signaling molecules to modulate secondary metabolite biosynthesis (Figure 2). This figure highlights the key signaling pathways including ROS signaling, metabolite signaling, and redox signaling. Mitochondria may regulate the biosynthesis of secondary metabolites, modulating plants’ adaptation to changing environmental conditions through these mechanisms.

### 4.1. ROS Signaling Pathway

ROS are highly reactive molecules produced in mitochondria and other organelles, including superoxide, hydrogen peroxide, and hydroxyl radicals. As the major source of intracellular ROS, mounts of ROS are generated from mitochondria [27]. In some steps of the mitochondrial ETC, especially at complex I and complex III, electrons are transferred from NADH and FADH2 to oxygen. During this process, some electron leakage may occur, leading to ROS production. Mitochondrial ROS levels increase in response to perturbations in the ETC or cellular stress. Elevated ROS levels can activate the ROS signaling pathway, which is critical in regulating secondary metabolites biosynthesis.

The ROS signaling pathway influences secondary metabolite synthesis by regulating specific transcription factors. Table 2 lists plant WRKY, MYB, and NAC transcription factors involved in ROS-mediated regulation of secondary metabolites’ biosynthesis and their target genes. These transcription factors, activated by ROS, play a key role in plant responses to stress. Specifically, these activated transcription factors can regulate gene expression to affect secondary metabolite synthesis, thereby assisting the plant’s response to stress. ROS can activate key enzymes in various secondary metabolic pathways. Specifically, ROS can alter the conformation and catalytic activity of enzymes by oxidative modification of active sites. For example, ROS oxidizes the MPK3 kinase, leading to its phosphorylation and activation in *Arabidopsis* [28]. Activated MPK3 kinase subsequently phosphorylates and activates WRKY transcription factors, which in turn induce the expression of genes involved in phenylpropanoid biosynthesis (e.g., *PAL*) [29,30,31]. Hu et al. found that ROS in *Actinidia deliciosa* could improve the activities of antioxidant enzymes and activate the key enzymes in the phenylpropane metabolism pathway, thereby improving the synthesis of total phenols and anthocyanins [32]. Moreover, ROS influences the synthesis and action of key plant hormones, including abscisic acid, jasmonic acid, and auxin, which play important roles in secondary metabolism regulation [33,34,35]. For instance, pathogen attack triggers mitochondrial ROS production, which activates the jasmonic acid biosynthesis pathway in *Solanum lycopersicum* [36]. Jasmonic acid can induce the synthesis of lycopene, carotenoids, and phenols by activating the expression of the transcription factor SlMYC2 [37]. Lycopene and carotenoids can help *Solanum lycopersicum* reduce oxidative damage caused by pathogen infections, while phenolic compounds can directly inhibit the growth of pathogens. Similarly, mitochondrial ROS can activate the salicylic acid biosynthesis pathway in *Arabidopsis* [38]. Salicylic acid enhances glucosinolate synthesis by inducing the expression of MYB51 [39]. Glucosinolates are known for their antibacterial and insect-repellent properties, thereby significantly improving the plant’s disease resistance.

Moderate levels of ROS play an important role in cellular signaling and regulation. However, excessive ROS can induce oxidative stress, which generally suppresses normal cellular metabolic processes. When mitochondrial ROS are overproduced, the excess ROS typically serve as a negative signal to inhibit secondary metabolite biosynthesis. High concentrations of ROS induce oxidative damage to cellular components, including lipids, proteins, and DNA, thereby disrupting the normal metabolic processes of cells [40]. Secondary metabolites biosynthesis will be inhibited due to decreased energy and substrate availability. In addition, high concentrations of ROS can inactivate key enzymes in secondary metabolic pathways. The study by Deshi et al. found that reduced ROS levels helped maintain or enhance the activity of PAL; therefore, it can be speculated that accumulation of ROS may harm PAL activity [41]. Excess ROS can also oxidize essential cofactors, such as NADPH and CoA, which are vital for secondary metabolic reactions [42]. Another important suppressive mechanism is that excessive ROS can induce programmed cell death, including in cells that are capable of synthesizing secondary metabolites. It is important to note that while the overproduction of mitochondrial ROS often acts as a negative signal to inhibit secondary metabolites biosynthesis, excess ROS can also promote the accumulation of specific secondary metabolites. For example, the accumulation of anthocyanins can be beneficial to resist the overproduction of ROS caused by environmental stress in *Arabidopsis* [43]. The relationship between ROS levels and secondary metabolite biosynthesis is complex and context dependent.

**Table 2:** Plant WRKY, MYB, and NAC transcription factors involved in ROS-mediated regulation of secondary metabolites’ synthesis and their target genes.

Transcription Factor	Target Gene	Regulatory Activity	Secondary Metabolites	Species	References
OpWRKY3	*OpTDC*, *OpCPR*	enhance	Camptothecin	*Ophiorrhiza*	[44]
OsWRKY6	*OsF3H*	enhance	Flavonoid	*Oryza sativa*	[45]
CcWRKY25	*pAMT*, *AT3*, *KAS*	inhibit	Capsaicin	*Capsicum*	[46]
WRKY40	*PAL*, *4CL*, *FLS*	enhance	Flavonoid	*Cyclocarya paliurus*	[47]
AtrWRKY42-2	*AtrCYP76AD1*	enhance	Betalain	*Amaranthus*	[48]
JsWRKY51	*JsTPS*	enhance	β-ocimene	*Jasminum sambac*	[49]
SlWRKY73	*SlTPS7*	enhance	Terpene compound	*Solanum lycopersicum*	[50]
VrMYB3, VrMYB90	*PAL*, *4CL*, *F3′5′H*, *LDOX*, *F3′H*	enhance	Anthocyanins	*Vigna radiata* L.	[51]
CcMYB12	*CcC4H*, *CcCHS*, *CcCHI*, *CcF3H*	enhance	Flavonoid	*Carya cathayensis*	[52]
FhMYB21L1, FhMYB21L2	*FhTPS1*	enhance	Linalool	*Freesia hybrida*	[53]
OsMYB30	*OsPAL6*, *OsPAL8*	enhance	Flavonoid, anthocyanins	*Oryza sativa*	[54]
MYB44	*CHS*, *FLS*	inhibit	Flavonoid	*Lonicera japonica*	[55]
SlMYB75	*SlTPS12*, *SlTPS31*, *SlTPS35*	inhibit	δ-Elemene, β-Caryophyllene, α-Humulene	*Solanum lycopersicum*	[56]
EsMYB90	*PAL*, *CHS*, *CHI*, *DFR*, *ANS*, *UFGT*	enhance	Anthocyanin	*Eutrema salsugineum*	[57]
SmMYB98	*SmGGPPS1*, *SmPAL1*, *SmRAS1*	enhance	Tanshinone, salvianolic acid	*Salvia miltiorrhiza*	[58]
CmtMYB108	*PAL*, *FNS*	enhance	Bioactive flavone	*Citrus maxima*	[59]
MdNAC1	*MdMYB10*, *MdUFGT*	enhance	Anthocyanin	*Malus domestica* ‘Red Flesh’	[60]
AaNAC2, AaNAC3, AaNAC4	*AaTPS1*	enhance	Volatile terpene	*Actinidia chinensis*, *Actinidia arguta*	[61]
LcNAC002	*LcSGR*, *LcMYB1*	inhibit	Anthocyanins	*Litchi chinensis*	[62]
EjNAC3	*EjCAD*	enhance	Lignin	*Eriobotrya japonica*	[63]
MdNAC42	*MdMYB10*	enhance	Anthocyanin	*Malus domestica* ‘Red Flesh’	[64]
ANAC078	*PAP1*, *TT1*, *TT2*, *AtMYB12*, *AtMYB4*	enhance	Flavonoid	*Arabidopsis thaliana*	[65]

### 4.2. Metabolite Signaling Pathway

Intermediates and metabolites produced by mitochondrial metabolism can also function as signaling molecules to regulate secondary metabolite biosynthesis. A prominent example is the succinate signaling pathway. Succinate is an intermediate in the TCA cycle. Hypoxia, inhibition of the ETC at complexes III or IV, or an increase in electron supply can lead to the reduction of coenzyme Q10H2 to coenzyme Q10 in the mitochondrion, subsequently leading to the accumulation of succinate [66]. Succinate accumulation drives superoxide production at complex I by contributing reducing equivalents to the mitochondrial respiratory chain. Specifically, fully reduced flavin mononucleotide reacts with O2 upon elevated levels of succinate, leading to copious superoxide production at complex I [67]. Succinate-driven superoxide generation can affect secondary metabolite biosynthesis by modulating enzyme activities, triggering oxidative stress responses, and regulating transcription factors. Additionally, succinate accumulation activates the succinate dehydrogenase complex, which inhibits mitochondrial respiration and leads to a decrease in intracellular ATP levels. The decrease in intracellular ATP levels promotes the phosphorylation and activation of AMP-activated protein kinase (AMPK) through changes in AMPK conformation mediated by increased AMP concentration [68]. Subsequently, activated AMPK can enhance secondary metabolite biosynthesis by upregulating the expression of secondary metabolite biosynthetic enzyme genes. For instance, AMPK can activate the phenylpropanoid biosynthetic pathway by upregulating the expression of *PAL* in *Arabidopsis* [69]. Activated AMPK also affects certain secondary metabolites’ biosynthesis by increasing the availability of acetyl-CoA through the promotion of fatty acid β-oxidation [70].

Citrate is another intermediate of the TCA cycle that can influence secondary metabolites’ biosynthesis via multiple pathways. Mitochondria produce citrate through the condensation of acetyl-CoA with oxaloacetate in the TCA cycle [71]. When cellular energy is sufficient, the TCA cycle generates an excessive amount of citrate, which can be transported out by the citrate–oxaloacetate shuttle system, subsequently regulating the synthesis of secondary metabolites. First, citrate can modulate metabolic pathways by affecting the activity of certain key enzymes. Citrate can activate citrate synthase, promoting the conversion of citrate to isocitrate. Isocitrate affects intracellular energy production by participating in the TCA cycle and serves as a precursor for the synthesis of other metabolites, thereby influencing secondary metabolites synthesis. When citric acid accumulates, it binds to phosphofructokinase and inhibits its activity, thus reducing the production of glucose-6-phosphate. Since glucose-6-phosphate is a precursor for the synthesis of secondary metabolites such as coumarins [72], the synthesis of these substances is inhibited. Furthermore, citric acid can affect AMPK signaling pathways, which are critical for regulating the metabolic state of cells and the synthesis of secondary metabolites. Fan et al. found that the accumulation of citrate can significantly upregulate the transcription and phosphorylation levels of AMPK [73]. These activated AMPKs can regulate secondary metabolism in various ways.

### 4.3. Redox Signaling Pathway

Mitochondria are critical regulators of intracellular redox balance. The activity of mitochondria can affect the redox balance, which in turn impacts the biosynthesis of secondary metabolites. Firstly, the ETC’s activity inadvertently generates ROS, potentially leading to increased ROS levels when the chain is compromised or under cellular stress, thereby altering redox homeostasis. Moreover, mitochondria house several oxidoreductases such as NADH dehydrogenase, coenzyme Q-cytochrome c reductase, cytochrome c oxidase, superoxide dismutase 2 (SOD), and glutathione peroxidase [74]. These enzymes are essential for maintaining the redox balance within cells. Furthermore, mitochondria participate in redox-sensitive metabolic pathways, including β-oxidation of fatty acids, oxidative deamination of amino acids, and the TCA cycle. These pathways produce strong reducing agents such as NADH, NADPH, and FADH2, which are essential for redox reactions in various biochemical processes, thereby impacting the cellular redox state.

Alterations in redox balance can profoundly affect secondary metabolite biosynthesis via multiple mechanisms. Firstly, an optimal redox state is a fundamental requirement for mitochondrial functionality and cellular metabolism, which has significant implications for cellular energy production and secondary metabolite synthesis. Further, the redox state modulates the activity and concentrations of enzymes involved in secondary metabolic pathways. This modulation affects the structural integrity and catalytic performance of enzymes such as polyphenol oxidase (PPO) in the biosynthesis of phenolic compounds and peroxidases in lignin synthesis [75,76]. Additionally, the redox state also influences the expression of genes, including *PAL*, *PPO*, *SOD*, and *chalcone synthase (ChaS1 and ChaS3)*, potentially augmenting their abundance [77]. In *Lactuca sativa* L., the redox status was altered under chilling conditions. This redox status alteration promoted the accumulation of kaempferol and quercetin via upregulating the expression of *CHS*, *Chalcone Isomerase*, *Flavanone 3-Hydroxylase*, *Flavonol Synthase*, and *Flavone Synthase I* genes [78].

## 5. Molecular Mechanism of Mitochondrial Regulation of Secondary Metabolite Biosynthesis

Mitochondrial activity is influenced by various regulatory mechanisms, such as transcription factors and microRNAs. These regulatory mechanisms can affect multiple mitochondrial activities, thereby impacting the synthesis of secondary metabolites.

### 5.1. Discovery and Function of Transcription Factors and Regulatory Elements

Transcription factors are diverse proteins that bind to DNA regulatory elements to control gene transcription, playing a crucial role in the biosynthesis of secondary metabolites by regulating mitochondrial genes. Table 3 compiles some of the transcription factors associated with mitochondrial function and secondary metabolite regulation. As shown in Table 3, the transcription factor TFAM is vital to regulating mitochondrial biogenesis and maintaining mitochondrial function [79]. The transcription factor NRF1 can bind to the mitophagy receptor FUNDC1, thereby promoting mitophagy and regulating mitochondrial homeostasis [80]. Considering the intricate relationship between mitochondria and secondary metabolism, alterations in mitochondrial metabolic processes can significantly impact secondary metabolite synthesis. For instance, under hypoxia, FOXO3 competes with the transcription factor c-Myc for transcriptional targets, suppressing c-Myc’s function and resulting in decreased mitochondrial activity and slowed respiration [81]. Since mitochondrial respiration is responsible for energy production and ROS generation, inhibiting mitochondrial respiration may lead to alterations in energy supply through secondary metabolic pathways and influence their synthesis via ROS signaling pathways.

### 5.2. The Role of MicroRNA in Mitochondrial Regulation

MicroRNAs (miRNAs) are a class of important non-coding RNA molecules that play an indispensable role in the regulatory networks of mitochondria. MiRNAs regulate gene expression by binding to target mRNAs and suppressing their translation or promoting their degradation. MiRNAs usually bind to the 3′ untranslated region of target mRNAs to suppress translation and mRNA degradation. Mitochondrial-associated miRNAs extend beyond the regulation of mitochondrial DNA (mtDNA) and nuclear-encoded mitochondrial gene expression; they can also directly influence mitochondrial function and metabolic pathways. As summarized in Table 4, various miRNAs affect secondary metabolite synthesis by regulating mitochondrial function. For instance, miR-15b can bind to the mitochondrial *SIRT4*, downregulating its expression and leading to mitochondrial dysfunction and increased ROS [88]. Specifically, miRNAs can modulate the expression of genes involved in the mitochondrial respiratory chain. Bukeirat et al. reported that miR-34a impairs mitochondrial function by targeting and repressing the expression of the *Cytochrome c(CYCS)* gene. CYCS is a subunit of mitochondrial respiratory chain complex III, and its downregulation leads to a decrease in mitochondrial respiratory chain activity and reduces ATP production [89]. In addition, miRNAs can regulate the expression of genes involved in mitochondrial ROS production. Mark Ziemann et al. reported that MiRNA-101-3p downregulates ETC complex subunits *NDUFA8* from complex I and *ATP5B* from complex V gene expression, resulting in decreased mitochondrial respiratory chain activity [90]. The reduced respiratory chain activity, in turn, triggers an increase in mitochondrial ROS production, and the increased ROS acts as a signaling molecule that modulates secondary metabolism. MiRNAs also modulate the expression of genes involved in mitochondrial metabolism. For example, Hsu et al. found that miR-181a suppresses parkin, resulting in the accumulation of ZNF746 and suppression of PGC-1α expression, ultimately inhibiting mitochondrial biogenesis and oxidative phosphorylation [91]. As detailed in Table 4, the interactions between miRNAs and mitochondrial function underscore the critical role of miRNAs in influencing mitochondrial-associated metabolic states and secondary metabolite synthesis.

### 5.3. Effects of Mitochondrial Modification and Post-Translational Modification on Metabolic Regulation

Epigenetic modifications and post-translational modifications (PTMs) are crucial mechanisms that regulate the transcriptional and translational frameworks of mitochondrial genes. These modifications function as dynamic regulators, influencing the gene expression profiles and functional status of mitochondria, thereby playing important roles in cellular metabolism and secondary metabolite biosynthesis. Recent studies have highlighted the importance of various modifications including acetylation, phosphorylation, Small Ubiquitin-like Modifier modification (SUMOylation), and methylation that play crucial roles in regulating the expression and function of mitochondrial genes and proteins [112].

Acetylation is one of the most widely studied PTMs in mitochondria, involving the addition of acetyl groups to lysine residues of proteins. Acetylation can modulate the activity of various mitochondrial enzymes such as mitochondrial malate dehydrogenase, further impacting mitochondrial energy production and the TCA cycle [113]. Consequently, acetylation influences mitochondrial metabolism, thereby further impacting secondary metabolism. Phosphorylation refers to the addition of phosphate groups to proteins, which can lead to significant changes in protein function. In the context of mitochondrial regulation, phosphorylation can activate or inhibit enzymes participating in metabolic pathways, then influencing the flux of metabolites through these pathways. For example, the phosphorylation of the pyruvate dehydrogenase complex leads to inhibition of its activity, which in turn leads to reduced acetyl-CoA production from the TCA cycle, further affecting secondary metabolism that utilizes acetyl-CoA as substrate [114]. SUMOylation is a reversible process that involves the covalent attachment of small ubiquitin-like modifiers to lysine residues of target proteins [112]. SUMOylation of mitochondrial proteins has been shown to regulate mitochondrial function and dynamics, as well as the induction of mitophagy. Yu et al. have found that SUMOylation of isocitrate dehydrogenase activates its activity, resulting in increased NADPH production [115]. This provides more reducing equivalents for the secondary metabolic pathways. Epigenetics refers to heritable changes in gene regulation that do not involve alterations in the DNA sequence itself, including DNA methylation and histone modifications. These modifications can lead to changes in chromatin structure, consequently affecting the transcriptional accessibility of mitochondrial genes [116]. Such epigenetic regulation can have long-lasting effects on gene expression patterns, potentially influencing secondary metabolite biosynthesis.

## 6. Prospect

The significance of secondary metabolites in ecosystems renders their biosynthetic mechanisms a sought-after yet intricate field of study. Owing to the chemical diversity and structural complexity of secondary metabolites, their biosynthesis is influenced by an array of intrinsic and extrinsic factors, encompassing genetic regulation, environmental conditions, plant hormones, and biotic interactions. Among these factors, mitochondria play a pivotal role as the metabolic hub; hence, unraveling the mechanisms by which mitochondria regulate the biosynthesis of secondary metabolites holds great significance. The mechanisms by which mitochondria regulate secondary metabolism primarily involve direct regulation of secondary metabolite synthesis through the provision of energy, substrates, and reducing equivalents and indirect regulation of secondary metabolite synthesis via the transduction of relevant signals to the pathway. Despite recent advancements in this domain, our understanding of how mitochondria orchestrate secondary metabolism remains incomplete, with numerous regulatory mechanisms and signaling pathways awaiting further exploration. It is imperative to delve deeper into the specific regulatory mechanisms in mitochondria, particularly the interplay of signaling pathways within and outside the mitochondria. To this end, we propose the following insights on the future exploration of how mitochondria regulate the synthesis of secondary metabolites, based on the findings of previous studies.

### 6.1. Integration of Metabolomics and Transcriptomics

Metabolomics analysis can identify specific metabolites and metabolic pathways in mitochondrial metabolism that are related to secondary metabolism. For example, Cao et al. conducted metabolomics analysis on millet subjected to drought stress and found that citrate, a key metabolite in mitochondria, plays an important role in the synthesis of anthocyanins [117]. Zhao et al. conducted a metabolomic analysis of quinoa and found that acetyl-CoA is an important precursor for the biosynthesis of triterpenoid saponins [118]. Transcriptomics provides information on the expression levels of all RNA molecules in cells or tissues, including nuclear genes encoding mitochondrial proteins and transcription factors involved in the regulation of secondary metabolite biosynthetic pathways. It can identify key genes and the regulatory mechanisms associated with mitochondrial function and secondary metabolite biosynthesis.

Integrating metabolomics and transcriptomics data provides a powerful approach for comprehending the regulation of secondary metabolite biosynthesis by mitochondria. First, key mitochondrial metabolic pathways related to secondary metabolism can be identified by correlating differentially expressed genes in transcriptome data with metabolite changes in metabolomics data. It will help determine which mitochondrial metabolites serve as precursors or regulators of secondary metabolism. Second, analysis of transcriptome data can identify transcription factors involved in the regulation of secondary metabolite synthesis. This can reveal which transcription factors are upregulated or downregulated in response to mitochondrial signals, thus elucidating the connections between mitochondrial metabolism and secondary metabolite biosynthetic pathways.

### 6.2. Gene Editing and Transgenesis Technologies

Gene editing and transgenesis technologies have been of great value in investigating the regulatory mechanisms of mitochondria in secondary metabolism. The clustered regularly interspaced short palindromic repeats-CRISPR-associated proteins systems, as a powerful genome editing tool, is one of the most significant discoveries in this century. Researchers can achieve specific knockout or editing of genes associated with mitochondrial function by leveraging advanced gene editing tools such as CRISPR/Cas9. We can further identify key regulatory factors by observing the changes in the yield and composition of secondary metabolites after editing specific genes. Liu et al. used CRISPR/Cas9 to edit the mitochondrial gene *OsNUDX14* in *Oryza sativa* and found that its lignin synthesis was reduced [119]. Further analysis revealed that the OsNUDX14 protein may regulate lignin biosynthesis by downregulating the transcription levels of key genes in the lignin biosynthesis pathway, such as *OsPAL1*, *OsPAL2*, and *Os4CL3* [119].

The application of transgenic technology will further expand the research field. Transgenic techniques provide valuable tools for investigating the effects of specific genes on mitochondrial metabolic pathways. Xu et al. constructed a transgenic line of Arabidopsis thaliana that overexpresses AtPAP2 in mitochondria. They found that the application of AtPAP2 to mitochondria increased the activity of TCA enzymes, the number of electron carriers in the mitochondrial ETC, and the production of ROS [120]. Furthermore, it can be used to construct plant models with different mitochondrial functional characteristics. By introducing specific regulatory factors or signaling pathways, these transgenic plants will help to reveal the specific role of mitochondria in secondary metabolism.

### 6.3. Ecological Adaptation Research

Under the background of increasing global climate change and environmental pollution, plants are facing various environmental stresses, such as drought, salinity, and pests. These stresses not only affect plant growth and development but also have a profound impact on their metabolic processes. One study by Sánchez-Rodríguez et al. studied the cherry tomato plant under water stress and found that water stress can regulate the phenylpropanoid biosynthesis pathway and increase the content of quercetin and ferulic acid, thus protecting plants from damage caused by H2O2 produced in the cytoplasm [121]. Additionally, Sarker et al. found that under drought conditions, certain amaranth cultivars exhibited an increase in phenolic compounds, including flavonoids, benzoic acids, and cinnamic acids [122]. These increased phenolic compounds help amaranth cope with drought stress.

Mitochondria play a key role in the plant response to environmental stress, especially in regulating secondary metabolism. Mitochondrial function and metabolism exhibit remarkable plasticity in response to environmental stress conditions. For example, plants might enhance mitochondrial respiration under environmental stress to increase energy supply to fuel stress responses. Meanwhile, mitochondria can also modulate ROS production to regulate secondary metabolism used to combat stress. Therefore, it is of vital importance to explore the metabolic adaptation mechanisms of mitochondria under environmental stresses. We should investigate in depth how mitochondria regulate secondary metabolism under specific environmental stress conditions, such as drought, salt–alkaline conditions, or pests. In the case of *Populus ussuriensis*, mitochondrial-generated excessive ROS under drought stress enhanced lignin and anthocyanin synthesis through both direct and indirect pathways, which improved tolerance to drought stress [123]. Understanding the metabolic adaptation mechanisms of mitochondria under environmental stresses not only elucidates how plants endure adversity but also establishes a crucial theoretical foundation for agricultural production. Through genetic engineering or molecular breeding, it becomes feasible to selectively enhance plant resilience to specific environmental stressors.

## Figures and Tables

**Table 1:** The potential substrates that mitochondria may provide for plant secondary metabolite synthesis.

Substrates	Source	Related Secondary Metabolites	Reference
Acetyl-CoA	Fatty acid oxidation and pyruvate oxidation	Carotenoids	[11]
		Sterols	[12]
		Flavonoids	[13]
		Polyketide	[15]
		Squalene	[16]
Oxaloacetate	TCA cycle and pyruvate oxidation	Coumarins	[17]
		Anthocyanins	[18]
		Astaxanthin	[19]
α-Ketoglutarate	TCA cycle	Vindolinine	[20]
		Morphine	[21]
		Alkaloids	[22]
Fatty Acids	Fatty acid oxidation	Carotenoids	[14]
Nucleotides	Pentose phosphate pathway	Anthocyanins	[23]

**Table 3:** Transcription factors associated with mitochondrial function and secondary metabolite regulation.

Transcription Factors	Effect on Mitochondria	Impact on Secondary Metabolite Biosynthesis	Reference
PGC-1α	Promotes mitochondrial biogenesis and oxidative metabolism	Substrate and energy supply; redox regulation	[82]
TFAM	Regulates mitochondrial biogenesis and function	Substrate and energy supply; ROS signaling	[79]
NRF1	Promotes mitochondrial generation and function; regulates mitochondrial autophagy	Substrate and energy supply	[80]
HIF-1α	Regulates mitochondrial stress and degradation	ROS signaling; substrate and energy supply	[83]
FoxOs	Regulates mitochondrial mass and respiration	Energy supply; ROS signaling	[81]
CREB	Regulates mitochondrial protein expression and dynamics	Substrate and energy supply	[84]
PPAR γ	Regulates energy metabolism and mitochondrial function	Substrate and energy supply; redox regulation	[85]
ERRs	Regulates mitochondrial dysfunction and gene expression	Substrate and energy supply; redox regulation	[86]
HSF1	Regulates mitochondrial function and homeostasis	Substrate and energy supply	[87]

**Table 4:** MicroRNAs’ effect on biosynthesis of secondary metabolites by regulating mitochondria.

MicroRNA	Target Gene(s)	Region	Regulation	Regulation on Mitochondria	Effect on Biosynthesis of Secondary Metabolites	Reference
miR-15b	*SIRT4*	3′-UTR	Downregulation	Increases mitochondrial ROS production and decreases mitochondrial membrane potential	Mitochondrial dysfunction; ROS signaling	[88]
miR-26b-5p	*Mfn1*	3′-UTR	Downregulation	Disrupts mitochondrial metabolism and promotes apoptosis	Altered mitochondrial activity	[92]
miR-27b	*FOXO1*	3′-UTR	Downregulation	Improves mitochondrial redox state and function	Mitochondrial dysfunction	[93]
miR-29a	*CD36*	3′-UTR	Downregulation	Impairs respiratory chain activity	Mitochondrial dysfunction	[94]
miR-34a	*NDUFC2*, *H6PD*, etc.	3′-UTR	Downregulation	Decreases mitochondrial protein expression	Mitochondrial dysfunction	[95]
miR-124-3p	*FOXQ1*	3′-UTR	Downregulation	Induces mitochondrial dysfunction via suppressing Sirt4	Mitochondrial dysfunction	[96]
miR-130a-3p	*GJA1*	3′-UTR	Downregulation	Causes mitochondrial dysfunction	Mitochondrial dysfunction	[97]
miR-137	*MEF2A*	3′-UTR	Downregulation	Increases mitochondrial biogenesis and oxidative phosphorylation	Alterations in mitochondrial activity and content	[98]
miR-140	*PINK1*	3′-UTR	Downregulation	Induces mitochondrial dysfunction and increases ROS	Mitochondrial dysfunction	[99]
miR-145-5p	*AIFM1*	3′-UTR	Downregulation	Induces mitochondrial dysfunction and decreases homeostasis	Mitochondrial dysfunction	[100,101]
miR-147	*NDUFA4*	3′-UTR	Downregulation	Impairs respiratory chain function	Mitochondrial dysfunction	[102]
miR-181a/b	*TFAM*	3′-UTR	Downregulation	Inhibits the transcription and replication of mtDNA	Mitochondrial dysfunction	[103]
miR-210	*ISCU*	3′-UTR	Downregulation	Impairs mitochondrial electron transport	Alterations in ROS content and energy supply	[104]
	*GPD2*	3′UTR	Downregulation	Decreases mitochondrial respiration and ROS	Alterations in ROS content and energy supply	[105]
miR-214	*SIRT3*	3′-UTR	Downregulation	Induces mitochondrial dysfunction	Mitochondrial dysfunction	[106]
miR-218	*PRKN*	3′-UTR	Downregulation	Impairs mitophagy	Altered mitochondrial content	[107]
miR-372	*SLC25A12*	3′-UTR	Downregulation	Disrupts mitochondrial metabolism	Decreased mitochondrial metabolism	[108]
miR-484	*YAP1*	3′-UTR	Downregulation	Reduces mitochondrial ATP and mtDNA	Subject to mitochondrial dysfunction	[109]
miR-574	*FAM210A*	3′-UTR	Downregulation	Impairs mitochondrial protein expression and activity	Mitochondrial dysfunction	[110]
miR-874-3p	*VDAC1*	3′-UTR	Downregulation	Inhibit the release of mtDNA and mitochondrial metabolites	Energy and substrates supply	[111]

## Data Availability

Not applicable.
